# Association between carotid diameter and the advanced glycation endproduct N^ε^-Carboxymethyllysine (CML)

**DOI:** 10.1186/1475-2840-8-45

**Published:** 2009-08-06

**Authors:** Marcus Baumann, Tom Richart, Daniel Sollinger, Jaroslav Pelisek, Marcel Roos, Tatiana Kouznetsova, Hans-Henning Eckstein, Uwe Heemann, Jan A Staessen

**Affiliations:** 1 Department of Nephrology, Klinikum rechts der Isar, Technische Universität München, Munich, Germany; 2 Genetic Epidemiology Unit, Department of Epidemiology, Maastricht University, Maastricht, the Netherlands; 3 Studies Coordinating Centre, Division of Hypertension and Cardiac Rehabilitation, Department of Cardiovascular Diseases, University of Leuven, Leuven, Belgium; 4 Department of Vascular Surgery, Klinikum rechts der Isar, Technische Universität München, Munich, Germany

## Abstract

**Background:**

N^ε^-Carboxymethyllysine (CML) is the major non-cross linking advanced glycation end product (AGE). CML is elevated in diabetic patients and apparent in atherosclerotic lesions. AGEs are associated with hypertension and arterial stiffness potentially by qualitative changes of elastic fibers. We investigated whether CML affects carotid and aortic properties in normoglycemic subjects.

**Methods:**

Hundred-two subjects (age 48.2 ± 11.3 years) of the FLEMENGHO study were stratified according to the median of the plasma CML level (200.8 ng/ml; 25^th ^percentile: 181.6 ng/ml, 75^th ^percentile: 226.1 ng/ml) into "high CML" versus "low CML" as determined by ELISA. Local carotid artery properties, carotid intima media thickness (IMT), aortic pulse wave velocity (PWV), blood pressure and fetuin-A were analyzed. In 26 patients after carotidectomy, CML was visualized using immunohistochemistry.

**Results:**

According to the CML median, groups were similar for anthropometric and biochemical data. Carotid diameter was enlarged in the "high" CML group (485.7 ± 122.2 versus 421.2 ± 133.2 μm; P < 0.05), in particular in participants with elevated blood pressure and with "high" CML ("low" CML: 377.9 ± 122.2 μm and "high" CML: 514.5 ± 151.6 μm; P < 0.001). CML was associated fetuin-A as marker of vascular inflammation in the whole cohort (r = 0.28; P < 0.01) and with carotid diameter in hypertensive subjects (r = 0.42; P < 0.01). CML level had no effect on aortic stiffness. CML was detected in the subendothelial space of human carotid arteries.

**Conclusion:**

In normoglycemic subjects CML was associated with carotid diameter without adaptive changes of elastic properties and with fetuin-A as vascular inflammation marker, in particular in subjects with elevated blood pressure. This may suggest qualitative changes of elastic fibers resulting in a defective mechanotransduction, in particular as CML is present in human carotid arteries.

## Introduction

The quality of elastic fibers reflects a hallmark of cardiovascular aging and can be affected by the deposition of interstitial collagen, calcification, lipid peroxidation and glycoxidation. The latter are induced by advanced glycation end products (AGEs) which are generated by non-enzymatic glycation and oxidation of protein and reducing sugars [1]. Their formation is increased in the blood and tissues of diabetic subjects as a result of hyperglycemia and is implicated in diabetes-associated vascular stiffening [1,2]. N^ε^-Carboxymethyllysine (CML) is the major non-cross linking AGE, which acts via the receptor of AGE (RAGE), thereby stimulating proinflammatory action [3]. In atherosclerotic lesions CML is present in infiltrating cells, suggesting a role of CML in the development of vascular lesions potentially by glycoxidation of elastin fibers [2].

Circulating levels of AGEs have been measured and related to the degree of coronary arteriosclerosis in both diabetic [4] and non-diabetic [5] patients with coronary artery disease and also to impaired left ventricular function in patients with type 1 diabetes. Recently McNulty et al. observed that the concentration of plasma AGEs is significantly higher in hypertensive than in normotensive subjects and related to aortic stiffness independent of age and blood pressure [6]. Therefore they suggested that plasma AGEs may play a blood pressure independent role in vascular remodeling in essential hypertension, in particular as vascular remodeling is associated with inflammation [7,8] which reflects a major action of AGEs [9]. However, latter results needed to be confirmed in a strict normoglycemic group and furthermore need to be extended to carotid artery properties. Based on these observations we studied the relationship between plasma CML, carotid and aortic properties, in normoglycemic subjects. To investigate in how far CML is associated with vascular inflammatory processes fetuin-A was investigated as biomarker for vascular inflammation [10,11]. In this context we furthermore investigated whether fetuin-A explains carotid diameter changes. Additionally, we investigated the presence of CML in carotid arteries with atherosclerotic lesions to link systemic measures with local function and local CML deposition.

## Methods

Hundred-two subjects of the FLEMish study of ENvironment, Genes and Health Outcomes (FLEMENGHO) [12] involving a random sample of families living in a defined geographical area in northern Belgium were included in this study. The Ethics Committee of the University of Leuven and Munich approved the study, respectively. All participants gave informed written consent. The inclusion criteria for the Flemish cohort were participation in a prospective substudy, the age of at least 18 years, no antihypertensive or antihypertensive treatment and normoglycemia defined as fasting blood sugar below 6.7 mmol/l. The inclusion criteria for the immunohistochemistry were atheromatous alteration of the carotid artery in context with the clinical need of carotidectomy.

For at least 3 h before being examined, the participants refrained from heavy exercise, smoking, alcohol or caffeine-containing beverages. Trained nurses measured blood pressure and anthropometric characteristics. They administered a questionnaire to collect information about each subject's medical history, smoking and drinking habits, and intake of medications. Each participant's office blood pressure was the average of five consecutive readings. Elevated blood pressure was a systolic blood pressure above 140 mmHg and/or 90 mmHg diastolic or use of antihypertensive treatment. Body mass index (BMI) was weight in kilograms divided by the square of height in metres. N^ε^-Carboxymethyllysine (CML) plasma level of free CML and protein-bound CML was measured using an ELISA kit (Microcoat, Bernried, Germany), following the instructions of the manufacturer. Intra- and interassay variability were below 5%. The entire cohort was grouped according to the median of plasma CML (median: 200.8, 25^th ^percentile: 181.6, 75^th ^percentile: 226.1). The values below median are referred to as "low" and these above median as "high" CML, respectively. Blood glucose, total cholesterol, HDL, LDL, triglycerides, and serum creatinine were also measured in all subjects by routine laboratory methods. Fetuin-A was measured by commercially available ELISA according to the manufacturer protocol [13].

By means of a pulsed ultrasound wall-tracking system (Wall Track System; Pie Medical, Maastricht, The Netherlands), 3 trained researchers obtained vascular measurements at the common carotid artery 2 cm proximal of the carotid bulb. During the ultrasound examination, an automated oscillometric device (Dinamap 845; Critikon Inc, Tampa, FL, USA) recorded blood pressure at the upper arm at 5-minute intervals. As for the conventional auscultatory measurements, cuff size was adjusted to the circumference of the upper arm. Standard cuffs had an inflatable bladder of 12 × 24 cm [14]. As described elsewhere, the observers used applanation tonometry with a pencil-shaped probe (Millar Instruments Inc, Houston, TX, USA) and calibration to mean arterial pressure and diastolic blood pressure at the brachial artery to derive the local pulse pressure at the carotid artery. We computed cross-sectional compliance (CC) and the distensibility coefficient (DC) from the diastolic cross-sectional area (A), the systolic increase in cross-sectional area (ΔA), and the local pulse pressure (PP): CC=ΔA/PP and DC = (ΔA/A)/PP. A and ΔA were calculated from diameter (D) and the change in diameter (ΔD) as A=π(Δ(D/2)^2 ^and ΔA=πX[(D+ΔD)/2)^2^-πX(D/2), respectively. The intraobserver intrasession variability was <10% for the carotid measurements. The intraobserver intersession and interobserver intrasession variability were of the same order of magnitude.

The observers also determined carotid-femoral pulse wave velocity (PWV) from the length of the carotid-femoral segment and the transit time of the pulse wave. The carotid-femoral segment was the difference of the distances between the site of the carotid ultrasound measurement and the suprasternal notch and between the suprasternal notch and the site of the femoral measurement. We measured PWV using a high-fidelity SPC-301 micromanometer (Millar Instruments, Inc.) interfaced with a laptop computer running the SphygmoCor software, version 6.31 (AtCor Medical Pty Ltd, Sydney, Australia).

Immunohistochemical CML staining of a carotid artery with atherosclerotic lesions was performed in human material after carotidectomy [15]. Twenty six paraformaldehyde-fixed, paraffin-embedded tissue sections were cut (4 μm), dewaxed and rehydrated. After endogenous peroxidase blocking (endogenous enzyme block, Dakocytomation, Glostrup, Denmark), slides were incubated with a 1:100 dilution of CML biotine 0.34 mg/ml antibody, which was a kind gift by Dr. C. Schalkwijk (University Maastricht). This was followed by incubation with a 1:400 diluted secondary rabbit anti- mouse IgG HRP antibody (Serotec, Wiesbaden, Germany) and developed in diaminobenzidine tetrahydrochloride (DAB substrate kit for peroxidase SK-4100; Vector Laboratories, Burlingame, CA, USA) and counterstained with hematoxylin.

Statistical analyses were performed using SPSS software version 15.0 (SPSS, Chicago, IL, USA). Comparison between subjects with above and below the median of CML was performed by unpaired t-test or the χ^2^-test. The Spearman correlation coefficient was assessed for CML and the clinical properties in the whole cohort and the subgroup of normotensive and hypertensive subjects. Results are expressed as mean ± SD. A p value of less than 0.05 was considered to be statistically significant.

## Results

Baseline characteristics of the Flemish cohort and according to the median plasma CML level of the cohort (median: 200.8, 25^th ^percentile: 181.6, 75^th ^percentile: 226.1) are given in Table 1. Apart from plasma CML values no significant difference was present between the "low" and "high" CML group, in particular no differences in fasting blood glucose and lipid profile. Thirty-three subjects had elevated blood pressure level, including 20 with "low" CML and 13 with "high" CML.

**Table 1:** Baseline Characteristics according to CML (n = 102, mean ± SD)

**Characteristic**	**All**	**CML ≤ Median**	**CML>Median**
		**(n = 51)**	**(n = 51)**
Age (y)	48.2 ± 11.3	50.2 ± 11.7	46.8 ± 10.7
Male, female	53/49	26/25	27/24
BMI (kg/m^2^)	25.8 ± 3.7	25.8 ± 3.7	25.8 ± 3.6
Smoking status	71/31	36/15	35/16
(nonsmokers/smokers)			
Brachial SBP (mmHg)	128.4 ± 14.9	128.1 ± 13.2	129.0 ± 16.9
Brachial DBP (mmHg)	82.7 ± 10.5	83.1 ± 9.3	82.4 ± 12.0
Brachial PP (mmHg)	45.8 ± 10.2	45.0 ± 10.0	46.6 ± 10.2
Cholesterol (mmol/L)	5.6 ± 1.0	5.5 ± 1.0	5.8 ± 0.9
HDL (mmol/L)	1.4 ± 0.3	1.3 ± 0.3	1.4 ± 0.3
LDL (mmol/L)	3.4 ± 0.9	3.3 ± 0.9	3.6 ± 0.9
Triglycerides (mmol/L)	2.5 ± 1.4	2.6 ± 1.6	2.2 ± 1.0
Glucose (mmol/L)	5.0 ± 0.7	4.9 ± 0.7	5.0 ± 0.8
Creatinine (μmol/L)	92.0 ± 17.1	93.0 ± 16.0	92.3 ± 17.8
Fetuin (μg/ml)	108.7 ± 52.3	100.8 ± 48.8	115.9 ± 55.2
CML (ng/ml)	210.4 ± 43.7	**183.0 ± 12.1**	**240.7 ± 46.1**

CML median: 200.8 ng/ml, Student's t-test and Mann-Whitney-U test where appropriate **Bold: *P *< 0.05**

CML: N^ε^Carboxymethyllysine, BMI: body mass index, SBP: systolic blood pressure, DBP: diastolic blood pressure, PP: pulse pressure.

**Figure 1:**
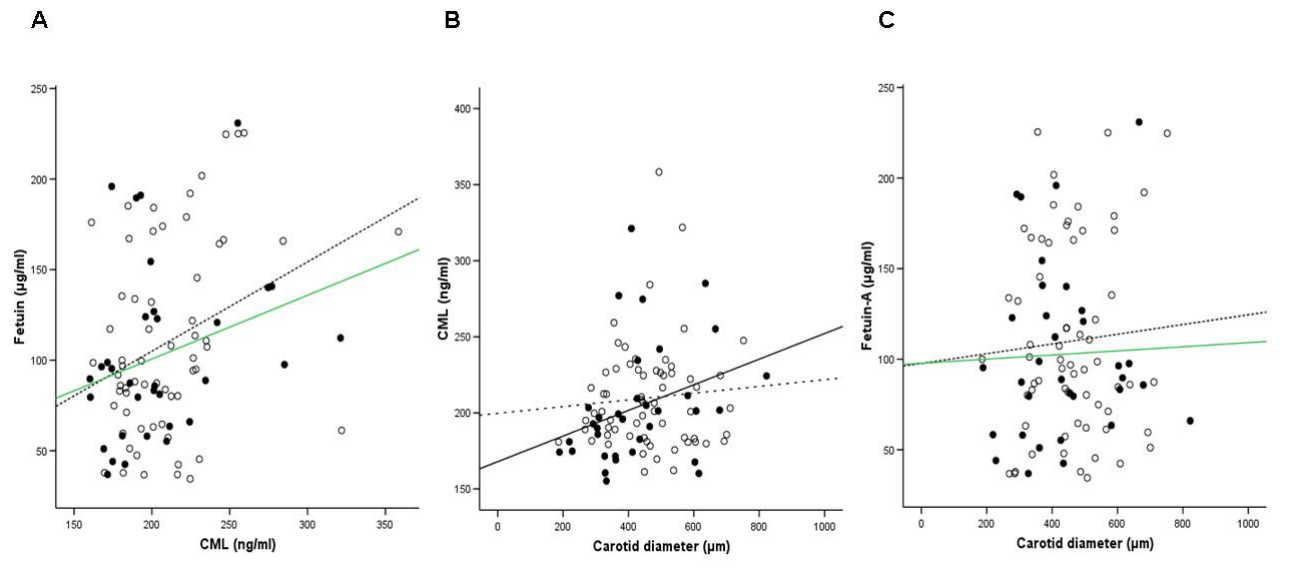
**Association between CML, Fetuin-A and carotid diameter**. CML and Fetuin-A are positively associated in hypertensive (open square) and normotensive (filled black square) subjects (A; r = 0.34 and r = 0.27, respectively). B demonstrates that CML is associated with carotid diameter restricted to hypertensive subjects (r = 0.42). C demonstrates that fetuin-A is not associated with carotid diameter. All correlations are given as calculated by Spearman correlation coefficient. Continuous lines indicate the regression of data represented by open circles and dotted lines those of closed circles.

Arterial properties according to the total cohort and according to the median plasma CML level of the cohort are given in Table 2. Carotid diameter was significantly larger in the "high" CML group (*P *< 0.05). PWV was not different between the "low" and "high" CML group. The bivariate Spearman association of CML and carotid diameter is for the normotensive cohort r = -0.01 (P = 0.92) and for subjects with elevated blood pressure r = 0.42 (P < 0.01; Table 3 and Figure 1). Additionally, the role of vascular inflammation was investigated with fetuin-A. CML is positively associated with fetuin-A (r = 0.28; P < 0.01). However Fetuin-A is in not associated with carotid diameter in any group (Figure 1).

**Table 2:** Arterial characteristics (n = 102, mean ± SD)

**Characteristics**	**All**	**CML ≤ Median**	**CML>Median**
		**(n = 51)**	**(n = 51)**
PWV (m/s)	6.8 ± 2.2	7.2 ± 2.6	6.5 ± 1.7
Common carotid artery			
IMT	0.82 ± 0.17	0.82 ± 0.17	0.81 ± 0.18
Diameter, μm	449.0 ± 130.7	**421.2 ± 133.2**	**485.7 ± 122.2**
Pulse pressure, mm Hg	45.0 ± 8.9	44.8 ± 9.2	45.6 ± 8.7
CC, mm^2^/kPa	0.91 ± 0.33	**0.85 ± 0.32**	**0.98 ± 0.34**
DC, 10^-3^/kPa	22.5 ± 8.4	21.4 ± 8.8	23.7 ± 8.0

CML median: 200.8 ng/ml, Student's t-test and Mann-Whitney-U test where appropriate **Bold: *P *< 0.05**

CML: N^ε^Carboxymethyllysine, PWV: pulse wave velocity, IMT: intima media thickness, CC: cross-sectional compliance, DC: distensibility coefficient

**Table 3:** Association between CML and clinical characteristics of the whole cohort, normotensive and hypertensive subjects

	**Total cohort**		**Normotension**		**Hypertension**	
	*r*	*P *value	*r*	*P *value	*r*	*P *value
**Age**	-0.17	0.10	-0.13	0.29	-0.17	0.34
**Sex**	-0.04	0.76	-0.15	0.24	0.19	0.30
**BMI**	0.05	0.60	0.01	0.99	0.17	0.33
**Smoking**	0.03	0.75	-0.02	0.88	0.05	0.78
**SBP**	0.02	0.81	0.09	0.46	0.30	0.09
**DBP**	-0.05	0.58	-0.04	0.73	0.17	0.34
**PP**	0.12	0.22	0.14	0.26	0.16	0.37
**Creatinine**	-0.10	0.37	-0.03	0.82	-0.11	0.54
**Glucose**	0.05	0.61	-0.08	0.55	0.13	0.46
**Cholesterol**	0.20	**0.05**	0.23	0.06	0.15	0.40
**HDL**	0.17	0.08	0.08	0.53	0.31	0.07
**LDL**	0.21	**0.04**	0.27	**0.03**	0.15	0.41
**Triglycerides**	-0.07	0.52	-0.04	0.71	-0.09	0.63
**Fetuin**	0.28	**0.01**	0.27	**0.03**	0.34	**0.04**
**PWV**	-0.12	0.23	-0.20	0.10	0.06	0.75
**Common carotid artery**						
**IMT**	0.08	0.45	0.15	0.25	-0.06	0.72
**Diameter**	0.18	0.07	-0.01	0.93	0.42	**0.01**
**Pulse pressure**	0.10	0.34	0.09	0.48	0.18	0.31
**CC**	0.12	0.26	-0.07	0.56	0.26	0.14
**DC**	0.11	0.30	0.01	0.99	0.21	0.23

Spearman correlation coefficient **Bold: P < 0.05**

CML: N^ε^Carboxymethyllysine, BMI: body mass index, SBP: systolic blood pressure, DBP: diastolic blood pressure, PP: pulse pressure, PWV: pulse wave velocity, IMT: intima media thickness, CC: cross-sectional compliance, DC: distensibility coefficient

Comparing subjects with normal and elevated blood pressure, no difference was apparent with respect to CML and carotid diameter. However, PWV was significantly elevated in the group with elevated blood pressure (6.5 ± 2.1 versus 7.5 ± 2.4 m/s; *P *< 0.05; Table 4).

**Table 4:** Baseline Characteristics according to blood pressure (n = 102, mean ± SD)

**Characteristic**	**Normal BP**	**Elevated BP**
	**(n = 69)**	**(n = 33)**
Age (y)	47.0 ± 10.5	50.6 ± 12.7
Male, female	36/33	17/16
BMI (kg/m^2^)	25.5 ± 3.5	26.3 ± 3.9
Non-Smokers/Smokers	49/20	22/11
Brachial SBP (mmHg)	**121.5 ± 8.7**	**142.2 ± 15.0**
Brachial DBP (mmHg)	**78.1 ± 6.9**	**92.0 ± 10.5**
Cholesterol (mmol/L)	5.6 ± 1.0	5.6 ± 0.9
HDL (mmol/L)	1.4 ± 0.4	1.4 ± 0.3
LDL (mmol/L)	3.4 ± 0.9	3.4 ± 0.9
Triglycerides (mmol/L)	2.4 ± 1.2	2.5 ± 1.6
Glucose (mmol/L)	5.0 ± 0.7	4.8 ± 0.8
Creatinine (μmol/L)	91.6 ± 15.8	91.6 ± 15.2
Fetuin (μg/ml)	110.0 ± 53.2	106.1 ± 51.4
CML (ng/ml)	214.2 ± 46.6	209.3 ± 40.7

PWV (m/s)	**6.5 ± 2.1**	**7.5 ± 2.4**
Common carotid artery		
IMT	0.81 ± 0.16	0.84 ± 0.19
Diameter, μm	458.5 ± 121.1	430.1 ± 148.1
Pulse pressure, mm Hg	**42.9 ± 7.7**	**49.2 ± 9.8**
CC, mm^2^/kPa	**0.96 ± 0.32**	**0.80 ± 0.32**
DC, 10^-3^/kPa	**24.0 ± 8.5**	**19.2 ± 7.3**

Student's t-test and Mann-Whitney-U test where appropriate **Bold: *P *< 0.05**, CML: N^ε^Carboxymethyllysine, BMI: body mass index, SBP: systolic blood pressure, DBP: diastolic blood pressure, PWV: pulse wave velocity, IMT: intima media thickness, CC: cross-sectional compliance, DC: distensibility coefficient

**Figure 2:**
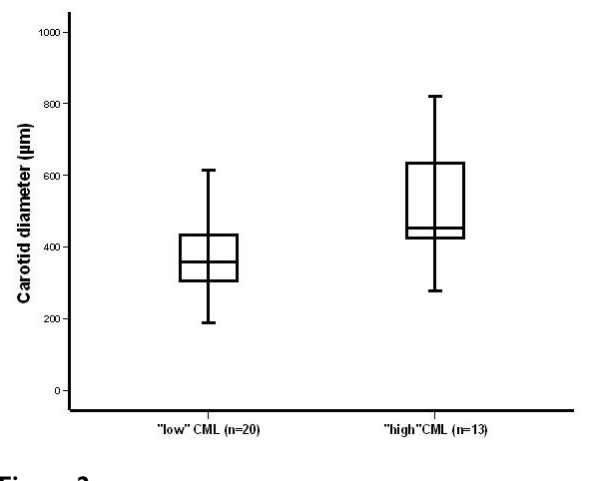
**Carotid diameter in subjects with elevated blood pressure (n = 33) according to the CML median**. Carotid diameter was significantly lower in 20 subjects with "low" CML (377.9 ± 122.1 μm) as compared to the 13 subjects with "high" CML (514.5 ± 151.7 μm; P < 0.001).

Subdividing the normotensive subjects according to the CML median no significant differences were apparent for carotid and aortic properties (data not shown). In contrast, subdividing the group with elevated blood pressure according to the CML median resulted in an increased carotid diameter in the 13 subjects with "high" CML (514.5 ± 151.6 μm) as compared to the 20 subjects with "low" CML (377.9 ± 122.2 μm, P < 0.001; Figure 2), while the elastic properties as cross-sectional compliance ("high" CML: 0.94 ± 0.33 versus "low" CML: 0.72 ± 0.29 mm^2^/kPa; P = 0.07) and distensibility ("high" CML: 21.2 ± 7.1 versus "low" CML: 18.0 ± 7.3 10^-3^/kPa; P = 0.20) were comparable between these two subgroups.

**Figure 3:**
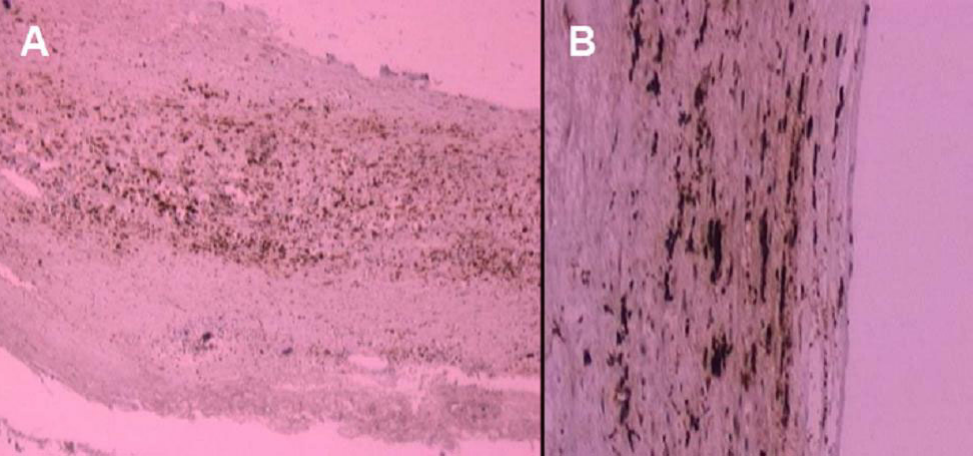
**Representative immunohistochemical staining of CML in the human carotid artery**. The magnification of **2A **(100×) and **2B **(400×) reveal CML positive cells in the subendothelial space.

The characteristics of the patients with carotidectomy are summarized in Table 5. Immunohistochemical staining of CML in the human carotid artery is shown in Figure 3. The magnification of **2A **(100×) and **2B **(400×) reveal CML positive cells in the subendothelial space. CML staining is present in atheromatous lesions and colocalizes with inflammatory cells.

**Table 5:** Characteristics of patients with carotidectomy

**Characteristic**	**Carotidectomy (n = 26)**
Age (y)	73.0 ± 8.4
Male, female	18/8
BMI (kg/m^2^)	26.6 ± 4.5
Smokers/Non-smokers	10/16
Brachial SBP (mmHg)	139.4 ± 14.3
Brachial DBP (mmHg)	77.4 ± 6.3
No. Antihypertensives	2.3 ± 0.9
Hyperlipidemia (%)	77
Statin (y/n)	21/5
Diabetes (%)	46
Insulin (y/n)	7/19
eGFR (ml/min)	65.3 ± 22.1
Present CVD (y/n)	6/20
CRP	0.65 ± 0.48

## Discussion

The main finding of this study is that carotid enlargement is apparent in context with elevated CML plasma levels which are still in the normal range. Moreover, the effect of CML on carotid enlargement is predominantly present in subjects with elevated blood pressure without changes in cross-sectional compliance or distensibility. Finally, CML was associated with fetuin-A as vasculo-inflammatory marker and was present in the subendothelial space of human carotid arteries.

CML is involved in vascular stiffening of type 1 diabetics as well as of hypertensive subjects [6,16]. Our study demonstrates that carotid enlargement is apparent in this cohort of normoglycemic subjects. This is particularly striking in subjects with hypertensive blood pressure values, where CML demonstrated an independent association with carotid diameter. Moreover, the shown effect is already present at a CML range below levels obtained in diabetes or renal insufficiency [17,18].

Although no prospective data exist on carotid diameter, Kawamoto et al. showed that the carotid artery diameter correlates with conventional cardiovascular risk factors including alcohol consumption [19], these findings suggest that the carotid artery diameter may reflect the ability of adaptive remodeling to the atherosclerosis before plaque formation. Moreover, increased carotid artery diameter as consequence of arterial stiffness limits the deterioration of the buffering capacity of the central artery [20]. Thus, CML can be an important factor during the development of atherosclerosis and may be relevant in clinical risk assessment. As atherosclerosis is driven by inflammation, we additionally assessed fetuin-A as marker for vascular inflammation [21,22]. In this context, we observed and association between fetuin-A and CML throughout the whole cohort. This suggests that CML potentially reflects the state of vascular inflammation [23]. This is further strengthened by our observation of CML in the subendothelial space of atherosclerotic human carotid artery material which is in line with previous descriptions in human aortic valves [24].

The mechanisms underlying this CML-related effect on carotid diameter cannot primarily be explained by an enhanced cross linking by AGEs as CML may affect the properties of the AGE-modified proteins but does not cause cross-linking in or between proteins [25,26]. Therefore, our findings suggest that the effect of CML on arterial diameter involves mechanisms other than cross-linking, such as ligation of the receptor for AGE (RAGE) [27]. RAGE is highly expressed in the endothelium of activated vessels [28]. Furthermore, CML is a ligand for RAGE [29]. Binding of circulating CML modified proteins to RAGE activates extracellular-regulated kinase 1/2 (ERK1/2), nuclear factor κ-B, secretion of proinflammatory cytokines, and modulates gene expression in several cell types, such as monocytes, endothelial cells, and vascular smooth muscle cells [30]. This may lead to changes in the production of extracellular matrix proteins, such as collagen and elastin structure, and alterations in vascular elasticity [31,32], as obtained by glycoxidation, and lipid peroxidation [24]. Because CML can be formed by glycoxidation and lipid peroxidation [2], the association of carotid diameter with CML may reflect the involvement of increased oxidative stress in arterial remodelling [33] which is further strengthened by the association with fetuin-A. However, as the serum concentration of soluble RAGE is in the picomolar range [34] whereas the CML molaritiy is in the micromolar range, it is questionable that sRAGE/CML binding occurs in vivo and has any biological effect. In this respect, serum CML is more likely an indicator of a dysfunctional cellular metabolism in the vascular wall in particular as we demonstrated CML deposition in atheromatous lesions of the carotid artery.

CML had no effect on carotid enlargement in subjects with normotensive blood pressure values. By contrast, in subjects with elevated blood pressure the CML plasma level characterized the extent of carotid enlargement without adaptive changes of the elastic artery properties. This may in turn lead to increased circumferential wall stress and consecutively result in a potentially defective mechanotransduction, that is, the control of smooth muscle cell growth and migration, and production of extracellular matrix in response to diameter enlargement [35]. Furthermore, a direct effect on elastin may be considered as CML co-localizes with elastin [36] and results in fenestration of elastic laminae [37].

In this cohort no effect of CML on PWV was observed. Therefore this study contrasts a recent observation describing CML as blood pressure independent factor in aortic stiffness [6]. However, there are important differences between both studies. Firstly, in the latter study AGE measurement was performed using a non-selective ELISA detecting other AGEs than CML as well, while in our study a specific ELISA for CML was used [38]. Secondly, blood glucose levels were elevated in the hypertensive group of McNulty et al., suggesting a more general glucose effect on both CML level and stiffness complicating the detection of the effects of AGEs alone. By contrast, our study excluded subjects with a fasting blood sugar of >6.7 mmol/l.

In summary, normoglycemic subjects with higher CML plasma levels are characterized by carotid enlargement without changes in elastic properties, in particular in elevated blood pressure. This may be associated with a qualitative change of elastic fibers and may lead to a defective mechanotransduction both explainable by local glycoxidation and lipid peroxidation induced inflammation.

## Competing interests

The authors declare that they have no competing interests.

## Authors' contributions

MB designed, coordinated and wrote the manuscript. TR, TK and JS generated the data of the Flemish cohort. JP and HE provided the human carotid arteries. DS and UH coordinated and wrote the manuscript. All authors read and approved the final manuscript.
